# Cross‐Sectional Imaging Useful in Melorheostosis

**DOI:** 10.1002/jbm4.10472

**Published:** 2021-02-18

**Authors:** Amelia Hurley‐Novatny, Apostolos H Karantanas, Georgios Z Papadakis, Timothy Bhattacharyya, Smita Jha

**Affiliations:** ^1^ Clinical and Investigative Orthopedics Surgery Unit, National Institute of Arthritis and Musculoskeletal and Skin Diseases National Institutes of Health Bethesda Maryland USA; ^2^ Medical Scientist Training Program, Carver College of Medicine University of Iowa Iowa City Iowa USA; ^3^ Department of Medical Imaging University Hospital, School of Medicine, University of Crete Heraklion Greece; ^4^ Advanced Hybrid Imaging Systems, Institute of Computer Science (ICS) Foundation for Research and Technology Hellas (FORTH) Heraklion Greece; ^5^ Metabolic Diseases Branch National Institutes of Diabetes and Digestive and Kidney Diseases Bethesda Maryland USA

**Keywords:** COMPUTED TOMOGRAPHY (CT), DRIPPING CANDLE WAX, MELORHEOSTOSIS/SOFT TISSUES, LERI'S DISEASE, MAGNETIC RESONANCE IMAGING (MRI), *MAP2K1*, SCLEROSING BONE DYSPLASIA, *SMAD3*

## Abstract

Melorheostosis is a rare disease of bone overgrowth that is primarily diagnosed based on imaging studies. Recently, the association of different radiological patterns of the disease with distinct genetic cause was reported. Several case reports have described the radiological findings in patients with melorheostosis. However, the added value of cross‐sectional imaging with CT and MRI beyond X‐rays has not been investigated. The aim of the current study was to investigate this existing gap in knowledge. Forty patients with melorheostosis seen at the National Institute of Health Clinical Center were included in the study, and all their imaging studies were analyzed. The sequence of interpretation was X‐ray followed by CT and then MRI. CT images were extracted from whole‐body 18F‐sodium fluoride positron emission tomography/CT studies. The information from CT reclassified the initial X‐rays based radiological pattern in 13 patients. Additionally, CT comprehensively identified joint involvement and disease extent. In 76% of patients (*n* = 29) who underwent MRI, additional findings were noted, ranging from soft tissue edema to identification of soft tissue masses and incidental findings. MRI did not provide additional information on skeletal lesions beyond CT scans. However, it revealed the extension of soft tissue ossification into ischiofemoral space in four patients who complained of deep gluteal pain consistent with ischiofemoral impingement syndrome. In addition, MRI revealed soft tissue edema in 20 patients, 9 of whom had bone marrow edema and periosteal edema in the tibias consistent with shin splints. These findings suggest that select patients with melorheostosis should be evaluated with both CT and MRI, particularly patients in whom the distribution of pain does not correlate with the anatomic location of the disease in plain radiographs. © 2021 The Authors. *JBMR Plus* published by Wiley Periodicals LLC. on behalf of American Society for Bone and Mineral Research.

## Introduction

Melorheostosis (Online Mendelian Inheritance in Man [OMIM] #155950) is a rare, nonhereditary skeletal dysostosis with hyperostotic lesions often associated with adjacent soft tissue changes.^(^ ^1^ ^)^ Its name originates from three distinct Greek words, “melos” = limb, “rheo” = to flow, and “osteosis” = bone formation, which alludes to its most commonly described radiological appearance: candlewax dripping down the long bones. The disease has a prevalence of about one per million,^(2^ ^)^ and is seen almost equally in men and women. In about 50% patients, it is clinically or radiologically evident by 20 years of age.^(^ ^3^ ^)^ We previously reported the largest published case series of 30 patients with the disease.^(^ ^4^, ^5^ ^)^

Although the appendicular skeleton is predominantly involved, skull and axial skeleton may be affected by the disease.^(^ ^5^, ^6^, ^7^ ^)^ In a radiological series of 23 cases, the lesions were classified in five patterns: (i) classic “dripping candlewax,” (ii) endosteal “osteoma‐like,” (iii) myositis ossificans‐like, (iv) osteopathia striata‐like, and (v) mixed.^(^ ^8^ ^)^ Interestingly, only five patients (22%) presented with the classic radiological appearance alone. We recently proposed the association of different radiological patterns of the disease with distinct molecular signatures.^(^ ^4^ ^)^ Our work points to an association of classic presentation with somatic mosaic activating mutation in MAP2K1^(^ ^4^ ^)^ and an association of endosteal pattern with somatic mosaic activating mutations in SMAD3.^(^ ^9^ ^)^ The radiological pattern of the disease in a patient can hence predict the genetic cause of the disease.^(^ ^4^, ^9^ ^)^ In addition to osseous lesions, soft tissue abnormalities may significantly contribute to patient symptoms and disease burden.^(^ ^5^, ^10^, ^11^ ^)^

Patients with melorheostosis typically present with pain or limitation of physical function, although the disease may remain symptomatically occult.^(^ ^5^, ^12^, ^13^ ^)^ There is no treatment for melorheostosis that significantly reduces disease burden. Available treatment options are largely symptomatic, and are typically treated with nonsteroidal antiinflammatory drugs or other pain relievers.^(^ ^3^, ^5^, ^14^ ^)^ Bisphosphonates have been used in these patients with mixed clinical outcomes ranging from mild improvement to no change in disease.^(^ ^3^, ^15^, ^16^, ^17^ ^)^ Receptor activator of NF‐κB ligand (RANKL) inhibitors have also been reported to result in clinical improvement of the disease.^(^ ^17^ ^)^ This is consistent with elevated RANKL/osteoprotegerin transcript ratio, an index of osteoclastogenic stimulus seen in melorheostotic bone.^(^ ^18^ ^)^ Surgical resection is generally advised only in select cases, wherein limitations of physical function significantly impair quality of life.^(^ ^3^ ^)^ It is unclear whether surgery on melorheostotic bone is beneficial. Long‐term follow‐up case studies have described recurrence at the surgical site, thus raising questions regarding the effects of surgery on the disease milieu.^(^ ^12^, ^13^, ^19^ ^)^ In addition to osseous lesions, a majority of patients also have soft tissue abnormalities, such as cutaneous changes,^(^ ^4^, ^5^, ^20^, ^21^ ^)^ soft tissue masses and ossification,^(^ ^22^, ^23^, ^24^, ^25^ ^)^ vascular malformations,^(^ ^26^ ^)^ and, rarely, malignancies.^(^ ^27^, ^28^, ^29^, ^30^ ^)^ In light of evolving treatment options, detailed information about disease burden would be useful in establishing a treatment plan. Osseous and soft tissue lesions may explain some of the clinical symptoms that these patients complain of, such as referred pain.^(^ ^31^ ^)^ In this largest reported radiological series, we sought to explore the additional information provided by cross‐sectional imaging modalities such as CT and MRI in patients with melorheostosis because there is minimal published literature detailing the added value of cross‐sectional imaging in melorheostosis.^(^ ^10^, ^24^, ^32^, ^33^, ^34^, ^35^ ^)^

## Patients and Methods

This study was approved by our institutional review board (Clinicaltrials.gov; NCT02504879) and conducted at the National Institutes of Health (NIH) Clinical Center in Bethesda, Maryland. Written informed consent was obtained from each patient. Forty patients with X‐ray appearance consistent with melorheostosis were evaluated with cross sectional imaging. Thirty‐nine patients had whole‐body CT scan performed in conjunction with 18F sodium fluoride (^18^F‐NaF) positron emission tomography (PET)/CT and 38 had focal MRI at the site of maximal skeletal disease burden as seen on whole‐body X‐rays. One patient had a technetium bone scan performed within the last 30 days prior to being evaluated at the NIH Clinical Center and was hence exempted from undergoing the ^18^F‐NaF PET/CT. Two patients declined MRI evaluation. MRI imaging included short tau inversion recovery, T1‐weighted (T1‐W) and proton density/T2‐W with spectral fat‐suppression sequences in various imaging planes. Specific parameters on the various MRI sequences varied because some of these examinations were performed at outside facilities. MRI was focused on areas of findings seen on plain radiographs.

Imaging findings were recorded by one senior musculoskeletal radiologist with 35 years of experience (AK) and a nuclear medicine/hybrid imaging specialist (GZP) in consensus. First, findings from plain radiographs were recorded for location as follows: monostotic, monomelic, or polyostotic. The classification was recorded as a modified Freyschmidt's classification^(^ ^8^ ^)^ (Table 1) with “osteoma‐like” being herein referred to as endosteal hyperostosis, myositis ossificans‐like as soft tissue ossification, and the terminology for all other classifications remaining unchanged. Any available repeat radiographs were evaluated for any change through the disease course.

**Table 1:** Modified Freyschmidt's Classification

Modified Classification	Freyschmidt's classification	Radiological features	Other diagnostic criteria
Endosteal hyperostosis	Osteoma‐like	Eccentric cortical thickening and increased density with lesion size >5 cm	Must involve 2 or more bones or have accompanying soft tissue abnormalities (e.g., scleroderma, subcutaneous fibrosis) above the involved skeleton
Classic	Classic candle wax	Hyperostosis of the cortical surface with candle wax‐like overgrowth on the inner or outer surface of bone	
Soft tissue ossification	Myositis ossificans‐like	Nodularly arranged osseous lesions within soft tissue in 2 or more regions adjacent to joint	Must be unilateral, cannot appear as structured lamellar bone, and cannot have history of trauma to the region or neurological deficit
Osteopathia striata‐like	Osteopathia striata‐like	Long, dense hyperostotic striations in cortex	striations must be unilateral
Mixed	Mixed	Any combination of 2 or more of the above radiographic patterns	

Note. Classifications made in this study were based Freyschmidt's proposed classifications,^(^ ^8^ ^)^ with terminology modified to better describe disease appearance.

CT images were extracted from the ^18^F‐NaF PET/CT studies in 39 patients and analyzed to confirm the plain radiographic findings and to make any possible additional comments on the number, location, and pattern of lesions. Ultimately, we relied upon CT for final classification of radiologic pattern of the disease because of its capability to assess the osseous structures without overlap and its utility for multiplanar reconstruction (MPR). MRI examinations were subsequently evaluated for the presence of bone marrow edema, soft tissue changes, and any intra‐articular lesions.

## Results

Of the 40 patients with melorheostosis, 39 were evaluated with CT and 38 underwent an MRI. The median age of patients was 49 years (range, 25–72 years). Twenty‐seven percent were women. Serial imaging was performed in nine patients. The mean imaging follow‐up from the first examination was 47.5 months (range, 0–159 months).

**Fig 1:**
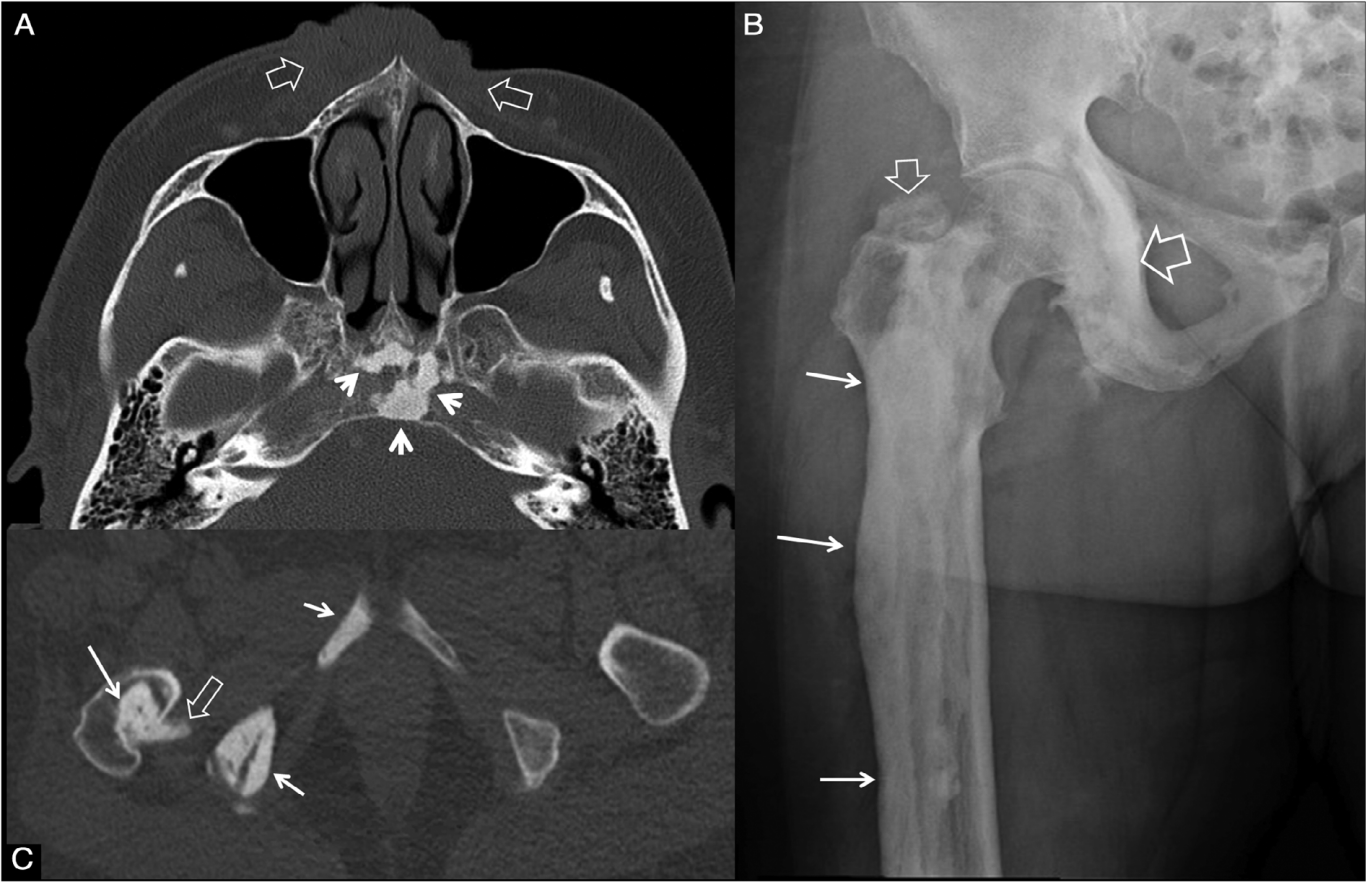
Value of CT in melorheostosis. A 60‐year‐old female patient (Melo‐13) with a follow‐up of 10 years after initial diagnosis. (*A*) Axial CT scan showing endosteal hyperostosis in the clivus (arrows) and subcutanous soft tissue lesions in the face on both sides of midline (open arrows). (*B*) Anteroposterior plain X‐ray of the right femur showing an extensive endosteal hyperostosis lesion in the proximal metadiaphysis of the femoral bone (arrows) and the ischiopubic bone (open arrow). A soft tissue ossification is shown above the greater trochanter (small open arrow). (*C*) Axial CT, bone window, in the pelvis, shows endosteal hyperostosis in the femoral (arrow) and ischiopubic bone (small arrows). Parosteal bone is extending into the ischiofemoral space, located into the quadratus femoris muscle (open arrow).

CT contributed to classification of disease location, especially when identifying whether the disease was monostotic versus monomelic or polyostotic (Fig. 1). Nine patients had monostotic, nine monomelic disease, whereas 23 had polyostotic bimelic disease (Table 2). Incorporation of information from CT scans reclassified four patients; three patients initially diagnosed as monomelic were reclassified as bimelic, whereas one initially diagnosed as polyostotic and bimelic from plain radiographs was reclassified as monomelic. There was no significant difference in prevalence of left‐sided (*n* = 15) versus right‐sided disease (*n* = 17). Nine patients had bilateral involvement. There was a predominance of lower (*n* = 14) versus upper extremity involvement (*n* = 8). One patient had both upper and lower extremity involvement without any lesions in the axial skeleton. Sixteen patients had axial lesions in addition to lesions in extremities, but none had axial involvement without involvement of an extremity. The pelvis was the most common site of axial involvement with nine patients affected. Few patients were noted to have lesions on the skull (*n* = 2), spine (*n* = 1), or ribs (*n* = 2).

**Table 2:** Value of CT Over Radiographs

Finding	Classification with CT, *n*	Classification with CT, %	Change with CT
Skeletal burden			
Monostotic	9 Upper limb 3 Lower limb 6	22	No change
Monomelic	9 Upper limb 3 Lower limb 6	22	1 Polyostotic ➔ monomelic
Polyostotic, bi/multi/melic	23	56	3 Monomelic ➔ polyostotic
Location			
Left alone	15	37	
Right alone	17	41	
Bilateral	9	22	
Upper limb(s) alone	8	20	
Lower limb(s) alone	14	34	
Upper and lower limbs, no axial	1	2	
Limbs and axial	16	39	
Axial alone	0	0	

Assessment with CT was found to change the Freyschmidt's classification performed initially with plain radiograph in 13 patients (Table 1 **)**. Furthermore, CT comprehensively elucidated joint involvement and disease extent, revealing intra‐articular extension in two patients (Melo‐29 and Melo‐34). Feet and pelvis were the two most frequent locations that CT modified the number and location of the lesions. Crystal deposition disease was found in tendons of two patients (hydroxyapatite in Melo‐35 and calcium pyrophosphate in Melo‐29).

**Fig 2:**
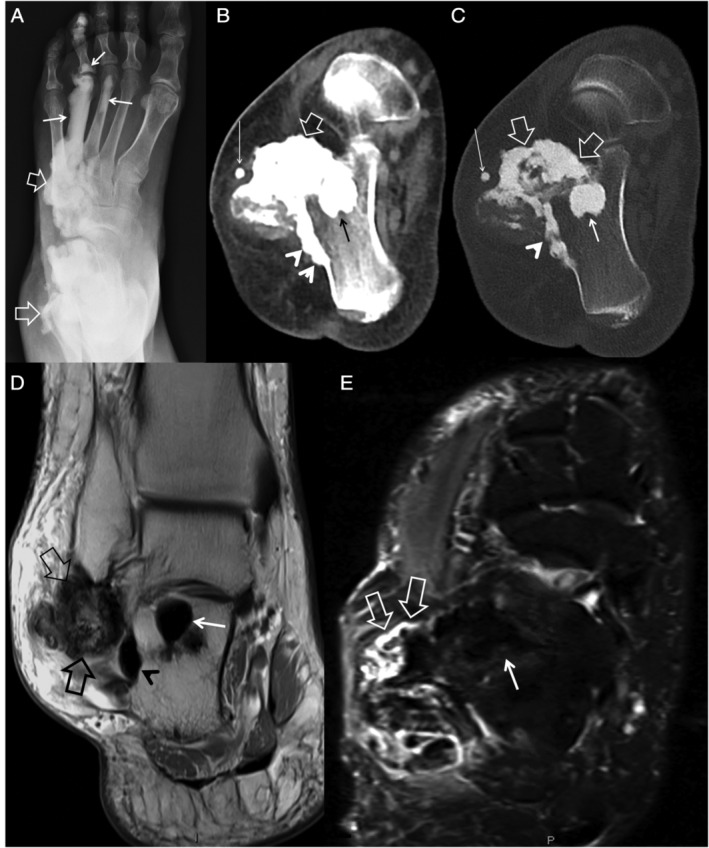
MRI in melorheostosis. A 42‐year‐old female (Melo‐4) patient with a follow‐up of 7 years after initial diagnosis. Plain radiograph (*A*) of the foot showing endosteal hyperostosis (thin arrows) and parosteal lesions (open arrows), not well‐defined because of overlapping structures. Axial CT images in the soft tissue (*B*) and bone window (*C*) show endosteal hyperostosis within the os calcis trabecular bone (arrows), the parosteal lesion on the lateral calcaneal cortex (arrowheads) and new bone formation of “dripping candle‐wax” pattern extending into the tarsal sinus (open arrows). Small soft tissue ossification (long thin arrow) is also shown. Coronal proton‐density–weighted (*D*) and axial fat‐suppressed T2‐ weighted (*E*) MR images showing the low signal endosteal hyperostosis (arrows), the parosteal hyperostosis (arrowhead in *C*), and a dripping candle‐wax lesion caudally to the lateral malleolus (open arrows) in greater detail as compared with CT. On the fat‐suppressed image, the dripping candle‐wax lesion is surrounded by a high signal‐intensity–reactive tissue (open arrows in *C*).

**Fig 3:**
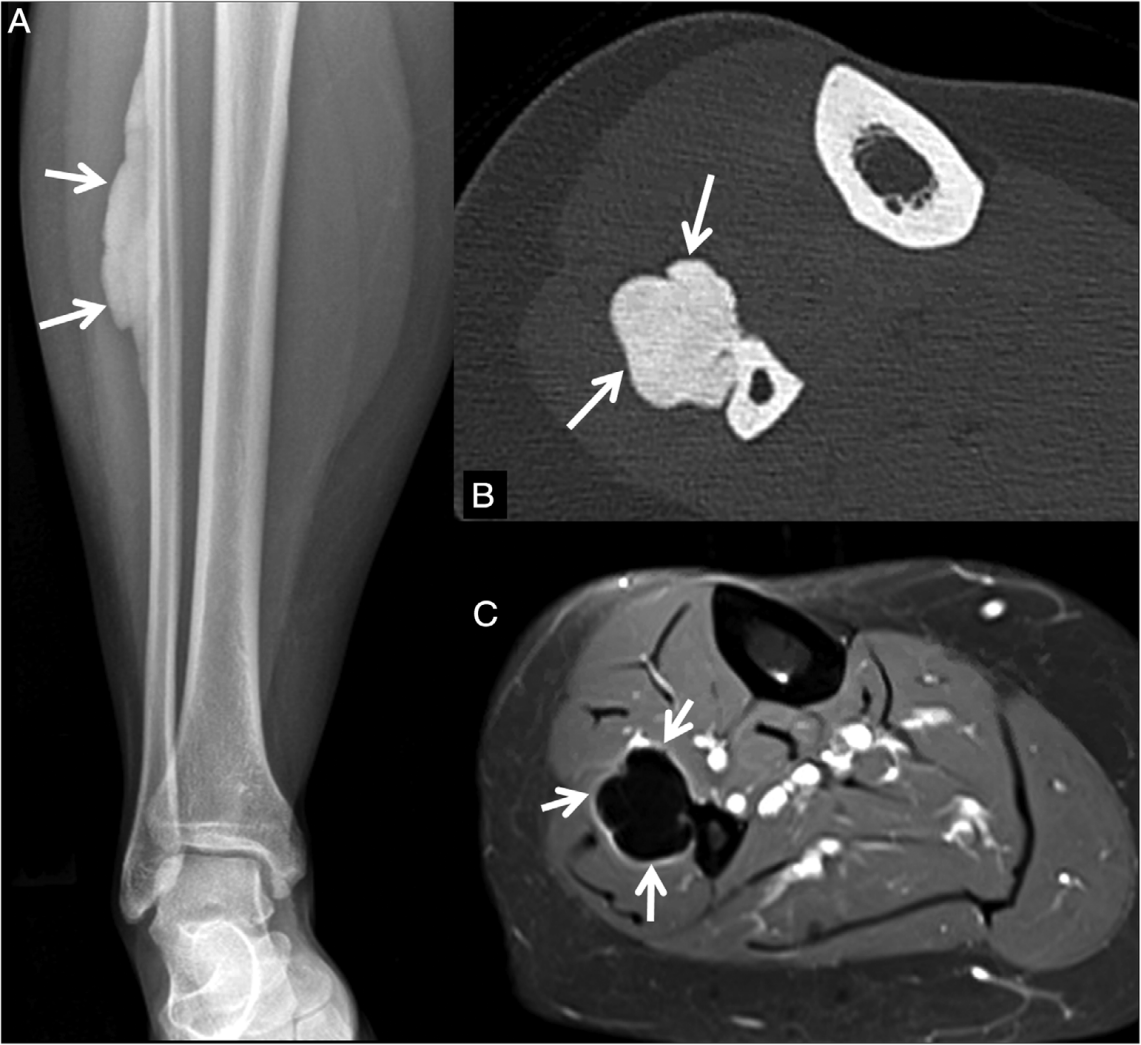
Parosteal lesion studied with radiographs, CT and MRI. A 53‐year‐old (Melo‐3) female patient with classic “dripping candle‐wax” lesion on the lateral aspect of fibular diaphysis, shown as compact bone (arrows) on plain anteroposterior X‐ray (*A*) and axial CT (*B*). Axial fat suppressed proton density‐weighted MR image (*C*) showing soft tissue edema surrounding the lesion (arrows).

**Fig 4:**
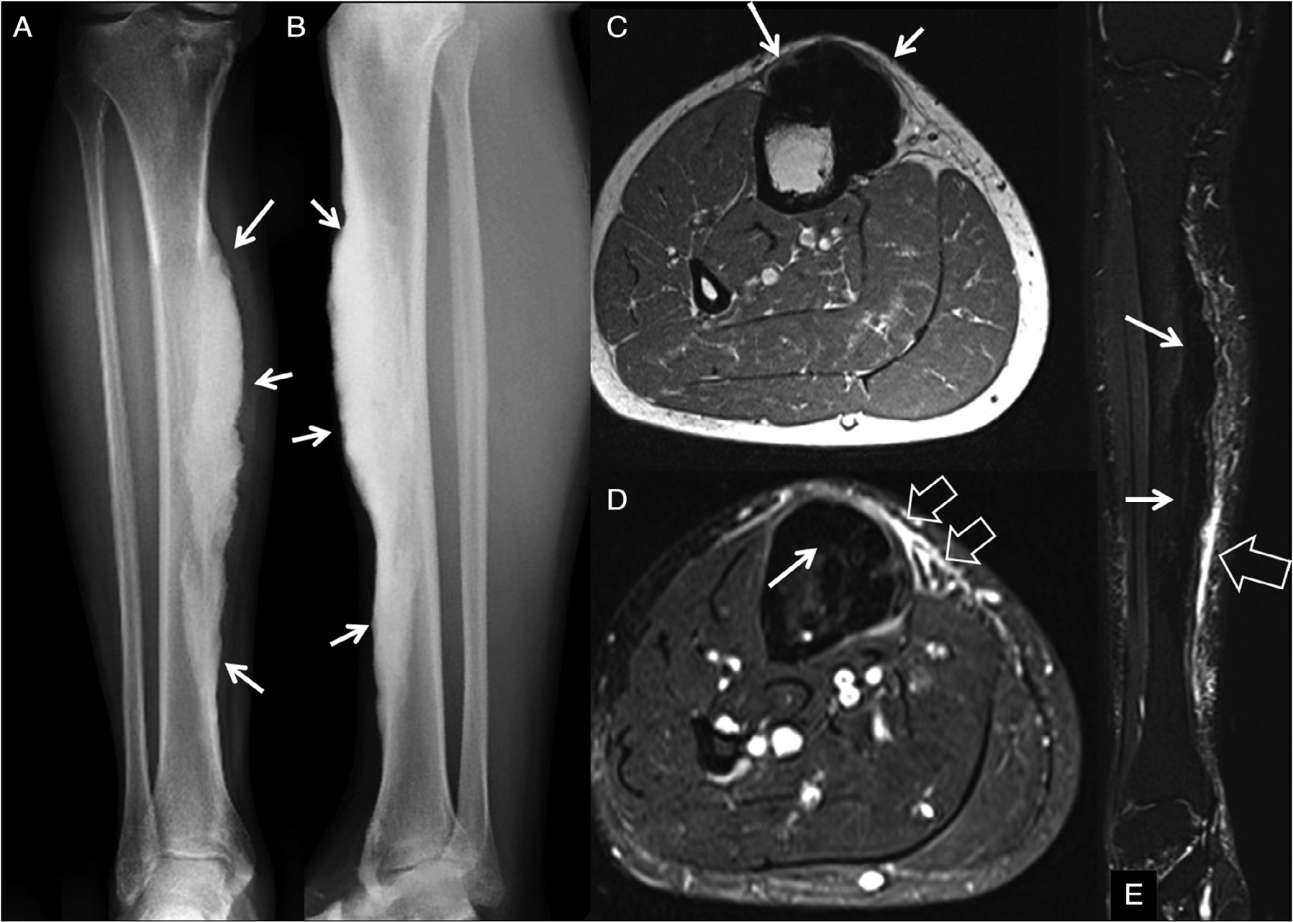
“Shin splints” in melorheostosis. A 46‐year‐old male (Melo‐2) patient with a follow‐up of 8 years after initial diagnosis. Anteroposterior (*A*) and oblique (*B*) plain X‐rays of the lower limbs, showing the dripping candle‐wax lesion located in the tibial diaphysis (arrows). The axial T1‐weighted (*C*), fat‐suppressed proton density‐weighted (*D*), and coronal short tau inversion recovery (*E*) MR images show the dripping candle‐wax lesion returning low signal on all pulse sequences (arrows). MRI shows in addition parosteal edema on the anteromedial aspect of the tibia similar to shin splints (open arrows).

**Fig 5:**
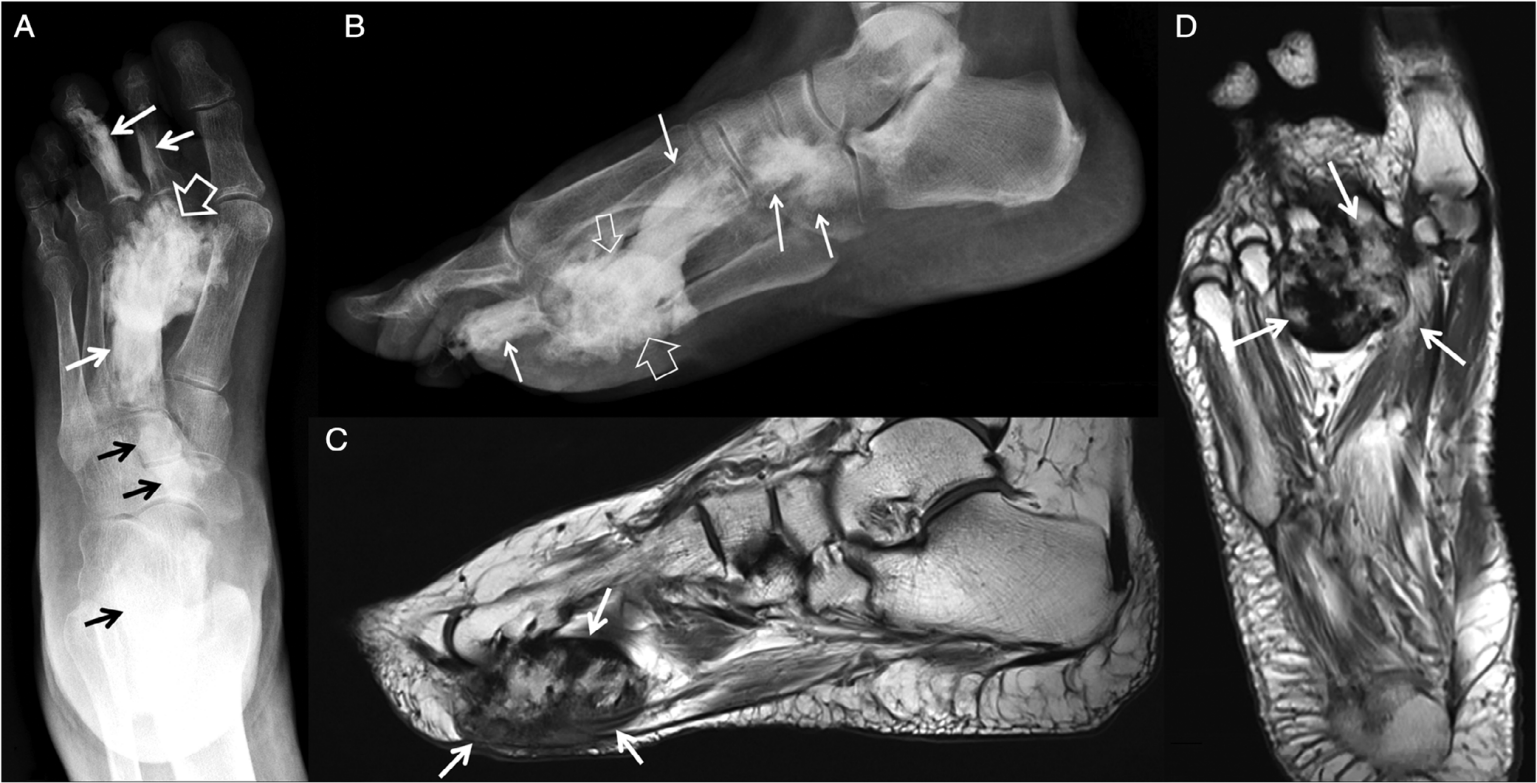
Various foot lesions in melorheostosis. A 63‐year‐old female patient (Melo‐41) showing on plain anteroposterior (*A*) and lateral (*B*) plain X‐Rays, endosteal hyperostosis lesions in the second proximal phalanx, in the third proximal and middle phalanx, in the third metatarsal, in the middle cuneiform and navicular bones in the midfoot, and distal tibia, and proximal talus (arrows). A large ossified lesion projects over midfoot and metatarsal bones (open arrows). Sagittal (*C*) and axial (*D*) T1‐weighted MR images show the location of the large soft tissue ossification plantar to distal metatarsals, displacing the muscles of the plantar aspect (arrows) in greater detail in comparison with plain radiographs.

MRI provided additional information on disease extent, particularly involvement of soft tissue (Figs. 2, 3, 4, 5). In 76% of patients (*n* = 29) who underwent MRI, additional findings were noted, ranging from edema to identification of more lesions and soft tissue masses. Generally, MRI did not provide additional information on skeletal lesions beyond CT scans. On all pulse sequences, osseous lesions showed low signal. MRI was useful in identifying extent and location of soft tissue ossification and associated soft tissue abnormalities in the affected limb. MRI confirmed the intra‐articular extension of the lesion in Melo‐29 and Melo‐34, already noted on CT. Four patients (Melo‐6, 10, 13, and 14) had extension of soft tissue ossification into the ischiofemoral space, all of them on the right side. Melo‐10 was noted to have ossification of the medial cruciate ligament (MCL) and soft tissue surrounding the knee, as well as fibrous changes within the joint, which explained her knee pain. In one patient (Melo‐30), the soft tissue ossification in the wrist showed a decrease in size in the 9‐year follow‐up imaging. MRI showed soft tissue in 19 and bone marrow edema in 8 patients. In six patients, both soft tissue and bone marrow edema were recorded. Nine patients showed periosteal edema in the anterior tibial surface corresponding to shin splints (Fig. 4). Three of these patients had bilateral lesions. Soft tissue edema was shown in eight patients, whereas bone marrow edema was shown in nine patients. Five patients showed a combination of two of the above findings, one with soft tissue and periosteal edema, another with soft tissue and bone marrow edema and three with bone marrow and periosteal edema. These findings may have been incidental in nature, but could explain at least some of the pain that these patients reported. MRI revealed extraosseous lesions not seen on CT, including suprapatellar fat pad edema (Melo‐12), Achilles xanthoma (Melo‐34), and muscular atrophy (Melo‐41) in one patient each. We did not find any change of the lesions over the follow‐up period of the study in the nine patients who had serial imaging.

## Discussion

This study is the largest series published to date regarding the significance of cross‐sectional imaging, specifically CT and MRI, in patients with melorheostosis. Through review of 41 cases from the NIH, we found that CT revealed additional lesions, which were not identified on plain radiographs, and better delineated the precise location of the lesions. Furthermore, we identified soft tissue abnormalities on MRI beyond those seen on CT imaging that could be clinically significant. These details are of particular significance in patients with involvement of the axial skeleton, in whom there is a greater concern for interference with the nervous system.

Although plain X‐ray alone may suffice to diagnose melorheostosis, CT scans help elucidate the precise involvement and extent of the disease. CT improved the identification of disease extent, including identifying additional lesions that were not immediately identifiable on plain radiograph. Some of these additional findings prompted a change in Freyschmidt's classification which can predict the genetic cause of the disease.^(^ ^4^, ^9^, ^18^ ^)^ In addition, a complete understanding of disease extent improves the understanding of patient's symptoms, such as pain or limitations in range of motion. Previous case studies have noted the usefulness of CT in the identification of additional lesions and/or understanding the disease extent.^(^ ^34^, ^36^, ^37^, ^38^, ^39^ ^)^ For example, CT commonly identifies intramedullary canal narrowing, which can result in pain.^(^ ^32^, ^38^, ^40^, ^41^ ^)^ Consistent with these reports, we noted the extension of melorheostotic lesions into ischiofemoral space, notably on the right side in all four patients, in the context of ischiofemoral impingement syndrome‐like symptoms, such as deep gluteal and/or hip pain. Furthermore, in patients in whom surgical management is being considered, CT is necessary for preoperative planning.^(^ ^24^, ^33^, ^38^, ^42^, ^43^, ^44^ ^)^

Melorheostosis, by definition, is a disease of the bone. However, it has become increasingly evident that there are, more often than not, surrounding soft tissue abnormalities associated with the disease.^(^ ^10^, ^45^, ^46^, ^47^, ^48^, ^49^ ^)^ These soft tissue abnormalities include fibrous changes, ossification, edema, cysts, vascular malformations, and rarely, malignant tumors.^(^ ^10^, ^32^, ^48^ ^)^ The extent of soft tissue involvement varies widely between cases, with some patients with debilitating soft tissue masses, while some are spared from any apparent soft tissue involvement. Soft tissue edema surrounding soft tissue ossification lesions can be attributed to friction of the muscles and tendons upon the space occupying lesion of melorheostosis. Soft tissue growths, fibrosis, and ossification can affect limb function through reduced range of motion and limb deformity.^(^ ^47^, ^48^, ^50^, ^51^ ^)^

Specifically, it may be important to monitor patients for joint involvement through MRI or CT to assess potential functional consequences, such as para‐articular or intraarticular ossifications that can result in joint pain, deformity, and interference with other joint structures. For example, ossification of the MCL, soft tissue edema, and fibrous changes of the knee were noted on MRI of Melo‐10, who reported knee pain. Other case reports also note severe joint involvement, such as glenoid labrum ossification, para‐articular—enhancing soft tissue masses, loose bodies within the joint, chondral lesions, and valgus deformity with permanent patellar dislocation.^(^ ^24^, ^47^, ^48^, ^50^, ^51^ ^)^ In all the aforementioned case reports, patients had severe disability of the limb that required surgical intervention. Additionally, because of the intricate nature of joints, identifying lesions that can affect function is prudent.

Although some of these findings are relevant and may affect disease management, other MRI findings are more incidental in nature and may be based on biomechanical differences in the limbs. Some of the incidental findings include shin splints, narrowing of the ischiofemoral space, and bone marrow edema. Shin splints often result from overuse, and present clinically with pain and local tenderness.^(^ ^52^ ^)^ We observed bilateral shin splints in three patients, and believe that this finding is related to biomechanical imbalances that cause improper stress distribution.^(^ ^52^, ^53^ ^)^ In patients presenting with shin pain, this could suggest a potential role for physical therapy and no weight‐bearing to reduce the pain. However, this recommendation can likely be made based on patients' symptoms alone. Bone marrow edema has been described in this study and others,^(^ ^32^, ^54^, ^55^ ^)^ and potentially contributed to the pain experienced by the patients in this study. Because of inadequate knowledge regarding the pathogenesis of melorheostosis, it is unclear if the edema results from inflammation or mechanical imbalances.^(^ ^56^ ^)^ Thus, though these findings may contribute to pain, they do not imply that an MRI is warranted to diagnose or treat all patients with melorheostosis. The presence of bone marrow edema supports the potential role of bisphosphonates in the treatment of melorheostosis,^(57^ ^)^ which has been described in several case reports.^(^ ^15^, ^16^ ^)^

Patients with melorheostosis involving the axial skeleton, primarily craniofacial bones and vertebrae, should be evaluated by both CT and MRI because of their proximity to the central nervous system. Specifically, CT should be conducted to gain a complete understanding of the disease extent, and MRI should be conducted to identify any associated soft tissue abnormalities. In several patients with disease involving the vertebrae, spinal stenosis and subsequent paralysis or neuropathy has occurred either at the time of diagnosis or years later.^10^, ^39^, ^43^, ^58^ ^)^ It would be prudent to monitor for progression and intervene early when appropriate. Although surgery in melorheostosis is generally recommended only in select patients, there have been limited reports of surgical management in cases of axial involvement. Surgical intervention in melorheostosis has been performed in patients when bone overgrowth or associated soft tissue abnormalities encroach on central nervous system structures^(^ ^6^, ^58^, ^59^ ^)^ or delicate facial structures,^(^ ^42^, ^60^, ^61^ ^)^ such as the nasal passages. In addition to CT, MRI is beneficial in these cases. Based on the preponderance of evidence that melorheostosis involves soft tissue as well,^(^ ^4^, ^7^, ^10^, ^45^, ^46^, ^47^, ^48^ ^)^ it is possible that neuropathy or occlusion of facial structures could be secondary to soft tissue masses. MRI offers the potential to provide a more complete understanding of the disease extent and guides clinical decision‐making regarding surgical resection. In this study, the pelvic bones were the most common site of axial skeleton involvement.

This study is the largest case series to comprehensively compare the additional benefits of cross‐sectional imaging in patients with melorheostosis. However, it has some limitations. First, MRIs were conducted at different locations, and thus vary in technique. Second, the CT data were extracted from CT performed with PE/CT studies, which is low‐dose, nondiagnostic CT performed for attenuation correction coregistration purposes. Third, a detailed correlation between clinical presentation and imaging findings was not performed in the current study.

In summary, our study is the largest case review of patients with melorheostosis that includes evaluation with cross‐sectional imaging techniques. Our findings confirm previous reports that CT and MRI are not needed for establishing a diagnosis of melorheostosis. However, the findings of the current study suggest that both CT and MRI of the site of the lesion are indicated when the X‐ray findings do not offer a complete explanation of the patient's symptoms. CT is the main imaging study for the exact location and characterization of a lesion, whereas MRI shows to better advantage the soft tissue and bone marrow lesions, which might explain patient symptomatology.

## Author Contributions

**Amelia Hurley‐Novatny:** Formal analysis; writing‐original draft; writing‐review & editing. **Apostolos Karantanas:** Conceptualization; formal analysis; methodology; supervision; writing‐original draft; writing‐review & editing. **Georgios Papadakis:** Methodology; writing‐review & editing. **Timothy Bhattacharyya:** Data curation; writing‐review & editing. **Smita Jha:** Conceptualization; data curation; formal analysis; investigation; methodology; supervision; writing‐original draft; writing‐review & editing.

## Conflict of Interest

The authors declare that there is no conflict of interests that could be perceived as prejudicing the impartiality of the research reported.

## Data Availability

The data that support the findings of this study are available from the corresponding author upon request.

### Peer Review

The peer review history for this article is available at https://publons.com/publon/10.1002/jbm4.10472.
